# Factors Associated with the Enjoyment of Physical Education Class for Children with Special Educational Needs

**DOI:** 10.3390/ijerph22050697

**Published:** 2025-04-28

**Authors:** Julie Dalgaard Guldager, Laura Mørk Emtoft, Line Nørgaard Remmen, Anette Lisbeth Bentholm

**Affiliations:** 1 Research Department, University College South Denmark, 6705 Esbjerg, Denmark; 2 Center for Teaching and Learning, University College Absalon, 4760 Vordingborg, Denmark; lme@pha.dk; 3 Department of Occupational Therapy, University College South Denmark, 6705 Esbjerg, Denmark; lnre@ucsyd.dk; 4 Department of Social Education, University College North, 9800 Hjørring, Denmark; anb@ucn.dk

**Keywords:** special education, physical education, primary school, secondary school, pleasure

## Abstract

Enjoying physical education class (PE) is important for children with special educational needs (SEN) due to its impact on their physical and mental health. However, there is a research gap concerning children with SEN and their enjoyment of PE. This exploratory study investigated how school children with and without SEN report enjoyment of physical education class and explored associated factors. A cross-sectional survey was conducted among school children with and without SEN. Both groups reported very high enjoyment of PE, but children without SEN had higher enjoyment. Among children with SEN, boys, those attending schools in East Denmark, and those engaged in leisure physical activity reported higher enjoyment levels, while children from larger schools consistently reported lower enjoyment levels. Understanding the differences in PE enjoyment between children with and without SEN is crucial for educators and policymakers to develop inclusive practices that ensure equitable experiences for all children, fostering a positive and supportive environment in school PE programs. Our findings highlight nuanced differences in PE enjoyment perceptions between children with and without SEN, emphasizing the need to consider diverse student characteristics when designing PE programs to enhance overall enjoyment.

## 1. Introduction

Enjoyment of physical education class (PE) in school is crucial for encouraging children to be active and improving their well-being [1,2]. It has been identified as an important predictor of PE participation [3] and level of fitness [4], and in a review of physical activity (PA) correlates for secondary school students in PE, enjoyment was consistently found to be positively associated with moderate to vigorous physical activity time in PE [5]. PE enjoyment is defined as a positively valenced emotion associated with feelings such as fun, joy, and pleasure directed towards PA [6].

The educational benefits claimed for participating in PE include physical, cognitive, social, and affective domains, where students can interact positively with peers and teachers, enhance cooperation skills, and exhibit empathy and respect [3,7,8,9]. A recent review on personal and social development within PE found that most studies reported a positive relationship between PE and social skills or life skills, as well as an increase in students’ perception of self-control, coping skills, managerial skills, and interpersonal skills [7]. Furthermore, Uddin et al. [1] found a positive association between participation in PE and levels of physical activity (PA) among adolescents. Given the diverse array of children in the public school system, including those with special educational needs (SEN), it is essential to consider how PE can be inclusive and beneficial for all students.

In international literature and educational policies and practices, various terms are used to denote children with SEN. This paper defines children with SEN as children with diverse social, intellectual or mental needs requiring additional specialized support [10], excluding physical special needs.

Children with SEN are often disproportionately excluded from PE [11,12], and they often receive special considerations that result in limited participation [13]. This exclusion is concerning as children with SEN exhibit lower levels of PA compared to their peers [14], putting them at risk for physical and mental health declines [15]. They also participate less in PA at school and during leisure-time [16,17], and they are more adversely affected by health conditions associated with physical inactivity [15,18,19] compared to their peers without SEN. Enjoyment of PE is particularly important for children with SEN, as research indicates that for children with disabilities, enjoyment of school-based PA is a significant factor for their perceived overall health [20]. A recent systematic review found a positive association between structured types of school PA, including PE, and mental health in children with SEN [21]. However, a recent umbrella review highlighted that students with disabilities often face restricted participation, marginalization, and exclusion in PE, frequently experiencing negative social interactions, such as bullying and social isolation [22]. Despite these challenges, more recent qualitative research suggests that positive experiences, such as positive social peer interactions, are also possible [22,23]. These mixed findings underscore the need to study the extent to which children with and without SEN enjoy PE and the factors associated with PE enjoyment in an inclusive education context where children with SEN primarily attend mainstream schools. By understanding these dynamics, we can better support the physical and mental health of children with SEN through more inclusive and enjoyable PE experiences.

Previous research has shown that school children in general have high PE enjoyment [24,25,26,27] (especially when feeling included [28]) and that boys tend to have more PE enjoyment than girls [25,29,30,31,32]. Further, PE enjoyment appears to decrease significantly with age [25,32,33]. For instance, a study examining children’s enjoyment of PE over three years found a significant decrease in PE enjoyment from the fourth to sixth grade. Additionally, enjoyment levels were notably lower among girls and students not participating in organized sports [25].

The current research on PE enjoyment and participation in leisure-time sports presents mixed findings, indicating a complex relationship that warrants further investigation. Cox et al. [34], in their longitudinal study, identified a correlation between students’ leisure-time physical activity and motivation-related experiences in PE, suggesting that behavioral aspects of PE experiences may be transferred to leisure activities. This finding aligns with Säfvenbom, Haugen, and Bulie [32], who found that adolescents participating in organized competitive youth sports reported significantly higher scores on attitudes towards PE. However, Brazendale et al. [35], did not observe a link between PE enjoyment and physical activity participation outside of school, highlighting a potential inconsistency in the literature.

As demonstrated, quantitative research on PE enjoyment among school children in general exists, but research on the experiences of PE among children with SEN is primarily qualitative. Thus, there is a notable research gap concerning quantitative studies on children with SEN and their PE enjoyment, to support the growing international body of qualitative research.

To fill this gap, this exploratory quantitative study aims to investigate how school children with and without SEN report their enjoyment of physical education class, and to explore which factors are associated with enjoyment of physical education class for school children with SEN. We hypothesize that school children with SEN report lower levels of enjoyment in physical education classes compared to their peers without SEN. Additionally, factors such as gender (boys), younger age, smaller school size, geographical location, and participating in leisure-time PA are hypothesized to be significantly associated with the enjoyment levels of PE for children with SEN.

### The Danish School Context

In Denmark, school is compulsory and free, and most children (78%) are enrolled in public school (22% in private schools) [36]. Children attend school for approximately 30–35 h weekly, and an average of 2 h pr. week are dedicated to PE.

In Europe, inclusive education models vary, with many countries integrating children with SEN into mainstream schools. Denmark follows this policy, with 6.5% of children with SEN in special education at dedicated institutions due to extensive support needs [37]. Consequently, most children with SEN participate in mainstream schools, including PE [38]. Danish legislation [37] registers school children by educational needs, not mental diagnosis, so this study focusses on special education needs rather than formal diagnosis.

In Denmark, the methodological guidelines for PE teachers emphasize a significant degree of pedagogical freedom, allowing educators to tailor their teaching methods to the needs and interests of their students. There are set objectives and knowledge areas for PE, but these are advisory rather than prescriptive, providing a flexible framework for instruction [39].

## 2. Materials and Methods

### 2.1. Procedure

This cross-sectional study used a quantitative survey conducted among school children in August 2023. An anonymous electronic questionnaire was administered in class with study assistants present to explain the survey and facilitate data collection. The analysis is part of the larger project “‘Moving’ physical education—for children with special educational needs in school and leisure-time”, which aims to provide positive PE experiences for children with SEN through co-teaching.

### 2.2. Participants

The study population included 498 school children aged 6 to 15 from 24 different school classes located at six schools in three different regions across Denmark.

Municipalities were contacted for potential participation in the larger project based on geographical location within socially disadvantaged areas [40], as students from these schools characterized by heightened socioeconomic disparity among students would likely yield more substantial benefits from project participation. The municipalities selected the schools in their area to join the project, without any specific criteria for inclusion or exclusion.

### 2.3. Ethics

This study followed Danish standards for the ethical conduct of scientific studies [41]. This study’s purpose was explained to the children, who were informed of their voluntary participation and assured of complete data anonymization, and parents received an information letter detailing the study.

### 2.4. Instrumentation

We explored the association of PE enjoyment with student sociodemographic characteristics (gender and age), school characteristics (size and placement), and student leisure PA. All research questions were closed, generated by the research team specifically for this study, and pre-tested with seven school children. The questionnaire covered the following areas:

#### 2.4.1. Enjoyment of Physical Education

The survey questions on PE enjoyment were inspired by the Physical Activity Enjoyment Scale [6] and the 2012 National Youth Fitness Survey [42]. Four questions measured children’s PE enjoyment: “*I enjoy participating in PE classes*”, “*When I have PE at school, I feel happy afterward*”, “*I think it’s fun to participate in PE classes*”, and “*When I participate in PE classes, it feels good in my body*”. Responses were on a 6-point Likert scale from 1 (strongly agree/green happy smiley) to 6 (strongly disagree/red unhappy smiley), with a “don’t know” option. This scale was chosen as research shows that children over seven can validly and reliably use scales with six faces [43,44,45]. The six-point scale is also effective in encouraging clear decisions rather than middle-ground choices [46], which is beneficial when surveying young children.

To facilitate group comparisons and simplify interpretation [47] for descriptive statistics, responses were dichotomized as agree (strongly agree, agree, somewhat agree) or disagree (strongly disagree, disagree, somewhat disagree). A sum score from 4 to 24 for the five questions was generated and recoded so that higher scores indicate greater PE enjoyment. The Cronbach’s alpha of the PE enjoyment scale was 0.85 in our sample.

#### 2.4.2. Sociodemographic Characteristics

Participants were asked about their gender (girl/boy/other) and age. Since less than 2% of the respondents identified as “other” in terms of gender, these responses were excluded from the analysis, as inclusion could compromise anonymity. To make comparisons more straightforward and to enhance the clarity and robustness of statistical analysis [48], age was divided into three groups of equal size (6–8, 9–11, 12–14 years old).

#### 2.4.3. School Characteristics

Information on school size was obtained from a Danish national register [49], and for statistical analysis, schools were divided into three groups of small (<300 children), medium (300–500 children), and large (>500 children) schools, since the average school size is 390 students [49,50].

#### 2.4.4. Leisure-Time Physical Activity

Participants were asked whether they attend any PA in their leisure-time (e.g., gymnastics, swimming, football, etc.) (yes/no).

#### 2.4.5. Special Educational Needs

Parents provided information about their children’s SEN status via a consent form linked to a unique ID number. They indicated (yes/no) whether their child “*has or is undergoing assessment for a neurodevelopmental disorder/psychological condition (e.g., autism, ADHD, anxiety, OCD, or other)*” and/or “*has undergone or is awaiting a psychological-pedagogical assessment*”, which is required in Denmark to determine the need for special educational support [38]. A positive response to any question classified the child as having SEN.

### 2.5. Data Analysis

Absolute and relative frequencies were used to describe the characteristics of the participating children in total and by group (children with/without SEN) (Table 1). Absolute and relative frequencies were used to describe the PE enjoyment, and 95% and Pearson’s chi-square was used to establish whether the differences between groups of children with and without SEN were significant (Table 2). Since the data were not normally distributed, the Mann–Whitney U test was applied to establish the difference between the means of the sum score for PE enjoyment (Table 2). Multivariate linear regression analysis was applied to determine the association between included variables and PE enjoyment (Table 3) among children with SEN. Results were presented as beta values (β) and 95% confidence intervals, and analyses were conducted separately for each independent variable (crude model 1) and concurrently (adjusted model 2). IBM-SPSS for Windows version 28 was used to conduct the statistical analysis.

## 3. Results

In total, 428 of 498 children (response rate, 85.9%) completed the questionnaire, resulting in an attrition rate of only 14.1%. Reasons for not participating included a lack of parental consent (*n* = 51) or absence on the day of data collection (*n* = 19). To ensure anonymity, eight children who had identified their gender as ‘other’ were omitted from the analysis. Thus, this study is based on responses from 420 children, of which 89 (21.2%) have SEN, and 331 (78.8%) have no such needs.

### Description of the Sample

Table 1 depicts information about the characteristics of the study population in total and stratified by the presence or absence of SEN among children. In the total study population, children were evenly distributed regarding gender (50% female). One third were between six and eight years old (30%), 60 percent were between nine and eleven years old, and 10 percent were between twelve and fifteen years old (mean age in total population was 9.44 years). More than half of the children (56%) attended a large school, 20% attended a medium sized school, and 25% attended a small school. More children attended a school in the regions of Northern Denmark (36%) and South Denmark (38%) than East Denmark (25%). Three quarters of children (74%) practiced PA in their leisure-time (e.g., gymnastics, swimming, football, etc.).

A statistically significant difference in gender distribution was observed among children with SEN compared to those without, with a higher percentage of children with SEN being boys (61%) compared to children without SEN (48%). Regarding the children’s age, more children with SEN (17%) were between the ages of 12 and 15 years compared to children without SEN (8.2%). Further, fewer children with SEN attended medium-sized schools (11%) compared to children with no SEN (22%). Finally, no difference was found in region or leisure-time PA between children with and without SEN.

Table 2 provides a detailed examination of children’s PE enjoyment, drawing distinctions between those with SEN and their peers without SEN. For all four aspects of PE enjoyment, a substantial majority of children in total, irrespective of SEN status, expressed a positive attitude towards participating in PE. However, for all four aspects, a statistically significant difference was observed between children with and without SEN, in that fewer children with SEN had positive attitudes compared to children without such needs. Finally, from the combined responses from all enjoyment-related questions, children in total scored a mean sum score of PE enjoyment of 19.95 (SD 4.177) from a possible score between 4 and 24. Both groups exhibited high PE enjoyment, but children with SEN had a significantly lower score (mean = 18.73) compared to those without SEN (mean = 20.29) (*p* = 0.029).

Table 3 shows the results of the linear regression analysis, investigating the factors associated with PE enjoyment among children with SEN. We found that boys exhibit significantly higher enjoyment levels compared to girls (β = 1.309, *p* = 0.002). No significant association between age group and enjoyment was identified. Children from large schools show lower PE enjoyment compared to children from small schools (β = −1.511, *p* = 0.008), and children from schools located in East Denmark show higher enjoyment compared to South Denmark (β = 1.504, *p* = 0.012). Finally, children engaging in leisure PA have significantly higher enjoyment levels than children who do not (β = 1.120, *p* = 0.026).

## 4. Discussion

This study found that children, regardless of their SEN status, reported high PE enjoyment, with children without SEN reporting more positive attitudes. Additionally, we identified factors influencing PE enjoyment for children with SEN, indicating that boys, children from schools in East Denmark, and those engaged in leisure PA reported higher enjoyment levels, while children from larger schools reported lower enjoyment levels.

Our study population had a balanced gender distribution, consistent with the gender distribution in the Danish national HBSC survey [51]. Consistent with previous research [52], we found a higher proportion of boys among children with SEN compared to those without SEN. Moreover, the prevalence of children aged 12–15 years was higher within the SEN cohort, which may reflect increased diagnoses with age [53]. In our cohort, 74% of children engaged in leisure-time PA, exceeding national averages (girls 68%; boys 72%) [51]. Additionally, a higher proportion of children attended larger schools compared to the national average school size [49]. Lastly, schools were strategically located across three municipalities, representing diverse regional areas within Denmark. Thus, caution should be made in generalizing our results to other populations.

Consistent with prior studies [24], our research shows that children in general reported high PE enjoyment. A noteworthy addition is our discovery that both children with and without SEN share this high enjoyment, which is crucial given its positive impact on their perceived health [20,21]. This may be because in PE, children, regardless of their needs, are in a different type of school activity and experiencing a break from academic instruction [54]. Furthermore, participation and advancement in PE are not contingent on performance, even though there are several goals in PE [55].

However, despite the overall high reporting of PE enjoyment as hypothesized, children with SEN reported lower levels of enjoyment compared to their peers. Similarly, Eversole et al. [56] found that children with autism spectrum disorders enjoyed leisure-time PA less than typically developing children. Hansen et al. [57] noted in their qualitative study that adolescents with intellectual disabilities sometimes feel bullied and fear exclusion in physical activities, mirroring potential challenges faced by children with SEN in school PE. Furthermore, children with SEN may experience physical challenges (e.g., coordination difficulties, sensory sensitivities) [58,59,60], social or cognitive challenges [61], or negative social interactions [22], which may make certain PE activities less enjoyable and more challenging for them. Our finding of lower PE enjoyment among children with SEN is concerning but not surprising, as they are often more likely to be excluded from PE activities [11,12]. This highlights the need for interventions to increase PE enjoyment for children with SEN.

We identified several factors influencing PE enjoyment. Previous research shows that boys tend to enjoy PE more than girls [25,29,30,31,32]. Our results indicate that, as hypothesized, this is also true for children with SEN. This finding is significant as it extends the understanding of gender differences in PE enjoyment to a population that faces unique challenges and barriers. DeVries et al. [62] found that Swedish girls with SEN have a higher academic self-concept than boys with SEN. This suggests that boys with SEN might view PE as a unique opportunity to participate and succeed, potentially leading to greater PE enjoyment compared to girls with SEN. The physical and social aspects of PE may provide boys with SEN a sense of achievement and inclusion that they might not experience as strongly in academic settings. However, it is important to consider that the dynamics influencing PE enjoyment among children with SEN may differ from those in the general population. Factors such as the level of support, the inclusiveness of the PE program, and the attitudes of peers and teachers towards children with SEN can significantly impact their PE experiences. Future research should explore these factors in greater detail to better understand the gender differences in PE enjoyment among children with SEN and to develop strategies to enhance PE experiences for all students.

In support of our hypothesis, we found that children with SEN from large schools enjoy PE less than those from small schools. This finding aligns with research by Zask et al. [63], who observed lower PA engagement during school recess in larger schools, suggesting stronger social inclusion in smaller schools. However, it is important to note that Zask et al.’s study focused on the general population of children, whereas our study specifically examined children with SEN. This distinction is crucial, as children with SEN may experience different social dynamics and barriers to participation compared to their peers in the general population. Evidence indicates that students in smaller schools feel more attached to their school [64,65]. This higher sense of social inclusion and attachment in smaller schools may influence PE enjoyment among children with SEN. The unique needs and experiences of children with SEN, such as the level of support and resources available, could be more effectively addressed in smaller school environments, leading to higher PE enjoyment. However, it is important to consider that our study’s results are based on a limited sample of seven schools, which may not fully capture the broader trends. The specific characteristics of the participating schools could have influenced our findings. Therefore, while our study suggests that school size impacts PE enjoyment for children with SEN, further research with a larger and more diverse sample is needed to support this proposition and to explore the underlying mechanisms in greater detail.

Unlike Barr-Anderson et al. [66], who found no geographical differences in PE enjoyment among American girls, our hypothesis on geographical differences was supported, as we observed such differences among students with SEN. This finding may be influenced by various factors, including demographic, contextual, cultural, or methodological differences between the studies. For example, the unique needs and experiences of children with SEN might be shaped by regional variations in educational resources, support systems, and community attitudes towards inclusion and physical activity. However, it is important to consider that our results are based on only seven schools, which may reflect school-specific differences rather than true geographical variation. The limited sample size and the specific characteristics of the participating schools could have influenced our findings. Therefore, while our study suggests geographical differences in PE enjoyment among students with SEN, further research with a larger and more diverse sample is needed to confirm these findings and to better understand the underlying factors contributing to these differences.

Previous research has predominantly focused on the general population of children, finding higher PE enjoyment among children engaged in leisure-time sports [32,34]. Our study extends this understanding by specifically examining children with SEN. Similarly, as hypothesized, we found an association between leisure-time PA and PE enjoyment for children with SEN. The association between leisure-time PA and PE enjoyment in this subgroup highlights the importance of considering diverse populations in research. Children with SEN may face unique challenges and barriers to participation in physical activities [11,12], making the positive link between leisure-time PA and PE enjoyment particularly noteworthy. This finding suggests that promoting leisure-time PA could be a valuable strategy for enhancing PE experiences for children with SEN, as it is possible that enjoying leisure-time PA increases motivation for PE, or vice versa. However, the causal relationship remains untested, and alternative explanations should be considered. For instance, it is possible that children who enjoy leisure-time PA have more positive attitudes towards physical activity in general, which translates into higher PE enjoyment. Conversely, children who enjoy PE might be more inclined to participate in leisure-time PA. Our finding also underscores the need for inclusive and adaptive PE programs that cater to the diverse needs of all students. By addressing this gap in the literature our study contributes to a more comprehensive understanding of the factors that influence PE enjoyment across different child populations.

### Limitations and Future Research

The results should be interpreted considering this study’s limitations. The main limitation is the cross-sectional design, which prevents exploring causality. Subjective assessments are challenging due to potential cognitive and motivational biases affecting validity [66,67]. Future research should consider longitudinal designs to ascertain the stability and evolution of these relationships over time.

Further, findings on factors influencing the PE enjoyment for children with SEN are based on a limited sample and should be considered exploratory, warranting further research. The proportion of children with SEN in our sample, despite being drawn from socially disadvantaged areas, matches that of general schools in Denmark [68].

Children’s self-reports may be biased due to selective memory or exaggeration of positive PE experiences [69]. This bias could be amplified by positively phrased survey questions and the positive response option being first. However, study assistants instructed students to answer honestly and explained the response options.

Furthermore, unlike prior studies using single-item measures to assess PE enjoyment [25,66,70], our study employs a multi-item measure, potentially enhancing reliability and validity. Although the Cronbach’s alpha of the PE enjoyment scale demonstrates high reliability, future research should further assess the reliability and validity of this measure. Additionally, it may be beneficial to investigate specific mechanisms or potential contributing factors underlying the observed associations.

Finally, it should be noted that all schools were in socially disadvantaged areas. Despite covering different regions, the study is limited to a single national context. Future research should replicate these findings in diverse international contexts to enhance generalizability and robustness.

## 5. Conclusions

Our study reveals that while most children, regardless of SEN status, express positive attitudes towards PE classes, there is a notable disparity with fewer children with SEN having positive attitudes compared to their peers without such needs. Specifically, among children with SEN, PE enjoyment is higher among boys, children from East Denmark, and those engaging in leisure physical activity, whereas children with SEN from large schools report lower PE enjoyment.

These findings contribute to the broader understanding of how diverse student characteristics influence PE enjoyment. They suggest that gender, regional differences, and extracurricular physical activity play significant roles in shaping attitudes towards PE among children with SEN. This underscores the need for theoretical models of PE engagement to incorporate these variables to better predict and explain variations in student experiences.

From a practical standpoint, our results highlight critical areas for intervention. Educators and policymakers should consider these diverse characteristics when designing PE programs to enhance enjoyment for all students. For instance, tailored strategies could be developed to support girls with SEN, children with SEN in large schools, and those not participating in leisure-time physical activity. By addressing these factors, PE programs can become more inclusive and supportive, fostering a positive environment that encourages all children to enjoy and benefit from physical education.

Future research should aim to validate our findings through longitudinal studies and explore additional factors that may influence PE enjoyment among children with SEN. Such studies could provide deeper insights into how these attitudes evolve over time and the long-term impact of targeted interventions.

Understanding these differences is crucial for educators and policymakers to develop inclusive practices that ensure equitable experiences for all children. By fostering a positive and supportive environment in school PE programs, we can help all students, particularly those with SEN, to thrive and enjoy the benefits of physical education.

## Figures and Tables

**Table 1:** Characteristics of all participating children and those of children with and without special educational needs.

		All		Special Educational Needs		No Special Educational Needs		*p*-Value
Overall *n*		417–420 ^a^		88–89 ^a^		329–311 ^a^		
Gender								**0.03**
	Girl	208	49.5	35	39.3	173	52.3	
	Boy	212	50.5	54	60.7	158	47.7	
Age								0.15
	6–8 years	124	29.5	23	25.8	101	30.5	
	9–11 years	254	60.5	51	57.3	203	61.3	
	12–15 years	42	10.0	15	16.9	27	8.2	
School size								**0.02**
	Small (<300 children)	103	24.5	27	30.3	76	23.0	
	Medium (300–500 children)	84	20.0	10	11.2	74	22.4	*
	Large (>500 children)	233	55.5	52	58.4	181	54.7	
Region								0.061
	Northern Denmark	153	36.4	23	25.8	130	39.3	
	South Denmark	161	38.3	41	46.1	120	36.3	
	East Denmark	106	25.2	25	28.1	81	24.5	
Leisure-time physical activity								0.136
	No	107	25.7	28	31.8	79	24.0	
	Yes	310	74.3	60	68.2	250	76.0	

^a^ Sample size for the different analyses diverges due to varying numbers of valid answers. * Indicates which group the *p*-value refers to; *p*-values in bold are statistically significant.

**Table 2:** Enjoyment of physical education classes of all participating children and that of children with and without special educational needs.

	Enjoyment of Physical Education							
		All		Special Educational Needs		No Special Educational Needs		*p*-Value
Overall *n*		393–413 ^a^		86–89 ^a^		307–324 ^a^		
I enjoy participating in physical education classes								**<0.001**
	Agree ^b, c^	367	89.3	68	77.3	299	92.6	
	Disagree	44	10.7	20	22.7	24	7.4	
When I have physical education at school, I feel happy afterward								**0.005**
	Agree	361	87.4	70	78.7	291	89.8	
	Disagree	52	12.6	19	21.3	33	10.2	
I think it’s fun to participate in physical education classes								
	Agree	364	89.2	71	81.6	293	91.3	**0.01**
	Disagree	44	10.8	16	18.4	28	8.7	
When I participate in physical education classes, it feels good in my body								
	Agree	345	85.6	67	77.0	278	88.0	**0.01**
	Disagree	58	14.4	20	23.0	38	12.0	
Enjoyment of physical education sum score (4–24 points)								
	Mean/SD	19.95	4.177	18.73	5.051	20.29	3.838	**0.029**

^a^ Sample size for the different analyses diverges due to varying numbers of valid answers. ^b^ The answering option of “don’t know” has been exempted from the analysis. ^c^ “Agree” = strongly agree, agree, somewhat agree; “Disagree” = strongly disagree, disagree, somewhat disagree. *p*-values in bold are statistically significant.

**Table 3:** Multivariate association between included variables and enjoyment of physical education classes among children with special educational needs (*n* = 86 *).

		Enjoyment of Physical Education					
		Crude—Model 1			Adjusted—Model 2		
		Beta	95% CI	*p*-Value	Beta	95% CI	*p*-Value
Gender							
	Girl	Ref.			Ref.		
	Boy	1.322	0.471–2.172	**0.002**	1.309	0.468–2.151	**0.002**
Age							
	6–8 years	Ref.			Ref.		
	9–11 years	−0.310	−1.269–0.650	0.526	0.368	−0.674–1.411	0.487
	12–15 years	−0.265	−1.971–1.441	0.760	0.726	−1.368–2.821	0.496
School size							
	Small (<300 children)	Ref.			Ref.		
	Medium (300–500 children)	−0.231	−1.499–1.037	0.720	−0.502	−2.499–1.496	0.621
	Large (>500 children)	−1.378	−2.436–−0.321	**0.011**	−1.511	−2.621–−0.401	**0.008**
Region							
	South Denmark	Ref.			Ref.		
	Northern Denmark	1.062	0.072–2.052	**0.036**	1.001	−0.497–2.518	0.188
	East Denmark	1.353	0.248–2.457	**0.017**	1.504	0.339–2.669	**0.012**
Leisure-time physical activity							
	No	Ref.			Ref.		
	Yes	0.992	−0.012–1.995	0.053	1.12	0.134–2.106	**0.026**

* The sample included participants with valid scores for all variables for enjoyment of physical education class. *p*-values in bold are statistically significant.

## Data Availability

The fully anonymized dataset that support the findings of this study are available on reasonable request from the corresponding author. The data are not publicly available due to privacy/ethical restrictions.

## Abbreviations

The following abbreviations are used in this manuscript:

PE	Physical education class
SEN	Special educational need
PA	Physical activity
